# Innovative Approach for a Classic Target: Fragment Screening on Trypanothione Reductase Reveals New Opportunities for Drug Design

**DOI:** 10.3389/fmolb.2022.900882

**Published:** 2022-07-04

**Authors:** Annarita Fiorillo, Gianni Colotti, Cécile Exertier, Anastasia Liuzzi, Francesca Seghetti, Alessandra Salerno, Jessica Caciolla, Andrea Ilari

**Affiliations:** ^1^ Institute of Molecular Biology and Pathology, Italian National Research Council, IBPM-CNR, Rome, Italy; ^2^ Department of Biochemical Sciences, Sapienza University, Rome, Italy; ^3^ Department of Pharmacy and Biotechnology (FaBiT), Alma Mater Studiorum, University of Bologna, Bologna, Italy

**Keywords:** fragment screening, trypanosomatid infection, trypanothione reductase, rational drug discovery, protein crystallography

## Abstract

Trypanothione reductase (TR) is a key factor in the redox homeostasis of trypanosomatid parasites, critical for survival in the hostile oxidative environment generated by the host to fight infection. TR is considered an attractive target for the development of new trypanocidal agents as it is essential for parasite survival but has no close homolog in humans. However, the high efficiency and turnover of TR challenging targets since only potent inhibitors, with nanomolar IC50, can significantly affect parasite redox state and viability. To aid the design of effective compounds targeting TR, we performed a fragment-based crystal screening at the Diamond Light Source XChem facility using a library optimized for follow-up synthesis steps. The experiment, allowing for testing over 300 compounds, resulted in the identification of 12 new ligands binding five different sites. Interestingly, the screening revealed the existence of an allosteric pocket close to the NADPH binding site, named the “doorstop pocket” since ligands binding at this site interfere with TR activity by hampering the “opening movement” needed to allow cofactor binding. The second remarkable site, known as the Z-site, identified by the screening, is located within the large trypanothione cavity but corresponds to a region not yet exploited for inhibition. The fragments binding to this site are close to each other and have some remarkable features making them ideal for follow-up optimization as a piperazine moiety in three out of five fragments.

## 1 Introduction

Leishmaniasis and trypanosomiasis are vector-borne zoonoses transmitted by parasites from *Leishmania* and *Trypanosoma* spp., belonging to the Trypanosomatidae family. It is estimated that, overall, these parasites affect 20 million people worldwide, causing 100.000 deaths every year (WHO, 2022b; WHO, 2022c; WHO, 2022d). These infections are included in the list of neglected tropical diseases (WHO, 2022a) and are diffused in some of the poorest countries in the world. However, leishmaniasis is endemic in the whole Mediterranean basin, and climate change is promoting the spreading of the insect vector, expanding the risk of human exposure to regions previously out of the range of the disease.

Currently available treatments are very unsatisfying due to poor efficacy, high toxicity, and increasing resistance. Despite the urgent need, no new drugs reached the market in the last 30 years and the number of compounds in a clinical trial is very low (Field et al., 2017), indicating that more efforts are necessary for the development of new classes of therapeutics.

Target-based drug discovery is a powerful approach to the early stages of drug development. The success of this method strongly relies on the careful selection of the target that must fulfill some requirements in order to assure efficacy and selectivity of the hits (Frearson et al., 2007).

Parasitic redox systems are a valuable source of promising targets since the maintenance of intracellular redox homeostasis is essential for parasite survival inside their hosts; moreover, these systems often depend on unique and nonredundant core redox enzymes (Salinas, 2013). In the case of the Trypanosomatidae, redox homeostasis relies on the dithiol trypanothione (TSH), a peculiar variant of glutathione (GSH) in which two GSH molecules are joined by the polyamine spermidine. TSH is kept in the reduced state by the action of the NADPH/FAD-dependent protein trypanothione reductase, homolog to the human glutathione reductase (GR).

Since its identification, TR has been considered a convincing target for drug discovery because of some attracting characteristics: 1) it is essential for parasite viability; 2) it differs from the homolog GR enough to allow for selectivity; and 3) it is druggable since it has been shown to be efficiently addressed by inhibitors (Battista et al., 2020). Moreover, the high sequence conservation of TRs from different species, reaching 100% for the TSH cavity, supports the possibility to develop a broad-spectrum drug against all trypanosomatid infections (Ilari et al., 2018).

However, TR has a significant limitation that must be taken into account in drug discovery. Indeed, previous studies showed that, in order to significantly affect parasite viability in culture, TR activity must be reduced by at least 90%, meaning that only inhibitors with sub-micromolar IC50 should be considered suitable drug candidates (Krieger et al., 2000). The identification of such inhibitors is particularly challenging considering that the substrate cavity is wide and solvent exposed, thus making it difficult to efficiently target it with small drug-like compounds (Patterson et al., 2011).

Considerable effort has been put together to identify new hits targeting TR, rationalize the interaction through structural characterization, and improve known scaffolds through SAR studies or structure-based design (Bernardes et al., 2013; Field et al., 2017; Battista et al., 2020), but despite some remarkable results (Patterson et al., 2011; De Gasparo et al., 2018; Turcano et al., 2018; De Gasparo et al., 2019; Turcano et al., 2020), none of the leads proposed has been yet promoted to preclinical trials, due to sub-optimal activity or toxicity issue. Most characterized inhibitors were found to target the TSH cavity, mainly the so-called mepacrine binding site (MBS) located at the entrance of the TSH cavity. More recently, an alternative structure-based strategy has been successfully explored, aimed to disrupt TR functional dimer by targeting a cavity located at the dimerization interface (Revuelto et al., 2021). However, it is very likely that other sites as well as other modes of inhibition not yet identified can be exploited for TR inactivation.

In this context, we decided to perform the first crystallographic fragment screening on TR, since we believe that it can make an important contribution to TR-targeted drug development. In fact, fragment screening is now well-established as a powerful approach to early drug discovery since it gives the unique opportunity to probe protein surfaces with limited collections of small molecules (150–250 Da) covering a large chemical space (Murray and Rees, 2009). Among the biophysical detection methods available for screening, crystallography has the advantage to identify the hits and define the binding mode in one single step. Therefore, it is possible to obtain a detailed mapping of the interaction hot spots of a target and to unravel unexpected mechanisms of inhibition. These results may be particularly useful to overcome the issues encountered so far with TR as a target. In fact, the detailed scanning of the TSH cavity as well as the identification of previously unexplored allosteric pockets has the potential to disclose new opportunities for lead design.

## 2 Experimental Methods

### 2.1 Protein Production and Crystallization

TR from *L. infantum* was produced as previously reported. TR from *T. brucei* (TbTR) was produced as previously reported (Turcano et al., 2020), with minor modifications. Briefly, the gene corresponding to TbTR (Uniprot ID: Q389T8) was cloned in the pET15b vector using the restriction site NdeI and XhoI to add an N-terminal His-tag. The construct TbTR-pET15b was transformed in *E. coli* BL21 (DE3) cells and expressed in LB medium by o/n induction with 0.5 mM IPTG (OD600 = 0.6) at 37°C. Cells were recovered by centrifugation, lysis was performed by sonication on ice (lysis buffer: 20 mM Tris-HCl pH 8, 300 mM NaCl, 5 mM imidazole, 0.1 mM PMSF, DNAse, 5 mM MgCl_2_). The soluble fraction was collected by centrifugation at 40,000 g for 20 min, clarified by filtration through a 0.2 μm syringe filter, and then applied to prepacked 5 ml HisTrap colums (Cytiva). The protein was eluted by a linear gradient against 500 mM imidazole (elution peak around 150 mM imidazole). The His-tag was removed by thrombin cleavage, 1 U of thrombin per mg of TbTR, overnight incubation at 4°C in 50 mM TRIS-HCl pH 8, 100 mM NaCl, 2 mM CaCl_2_. Thrombin was removed by Benzamidine-Sepharose 6B resin (Cytiva) and tag-free TbTR was further purified by reverse immobilized metal affinity chromatography (Protino Ni-TED resin, Macherey-Nagel). Finally, the buffer was exchanged in a centrifugal concentrator with 20 mM HEPES pH 7.5, the protein was concentrated at 12 mg/ml, frozen in liquid nitrogen, and stored at −80°C.

The crystallization condition suitable for screening at the Diamond Light Source XChem facility was identified by optimizing the conditions previously reported (Patterson et al., 2011). Little differences in the concentration of the precipitants led to two different crystal forms: plate-like monoclinic crystals with the tetrameric asymmetric unit (AU), same as reported in the original study, and tridimensional orthorhombic crystals with dimeric AU. The orthorhombic crystals have been considered more favorable since they resulted to be more resistant to mechanical stress and diffract consistently up to 1.6 Å; moreover, the dimeric AU, corresponding to the biological unit of TR, is more convenient than the tetrameric one for the inspection of the events identified by the Pan-Dataset Density Analysis (PanDDA). The selected crystallization condition is composed as follows: 22% 2-Methyl-2,4-pentanediol (MPD), 14% PEG3350, imidazole 40 mM pH 8. 50 mM NaBr must be added to the protein solution to get regular and well-diffracting crystals. Protein and crystallization solution was then shipped in dry ice to the XChem facility where crystallization plates have been set up in SwissCi 3-drops midi plates using 200 nl of protein solution plus 200 nl of crystallization solution.

### 2.2 Fragment Screening Experiment and Structure Solution

A total of 381 fragments from the DSI poised library (https://enamine.net/compound-libraries/fragment-libraries/dsi-poised-library) (500 mM stock concentration dissolved in DMSO) were transferred directly to TbTR crystallization drops using an ECHO liquid handler (50 mM nominal final concentration) and soaked for 1–2 h before harvesting. Data were collected at Diamond light source beamline I04-1. A total of 371 datasets were collected in the resolution range of 2.9 Å or higher with the majority being in the range of 1.6 Å to 2.2 Å.

Data processing was performed using the automated XChem Explorer pipeline (Krojer et al., 2017). Fragment hits were identified using the PanDDA algorithm (Pearce et al., 2017) followed by visual inspection. Refinement was performed using REFMAC (Murshudov et al., 2011). A summary of data collection and refinement statistics for all fragment-bound datasets and the reference dataset is shown in Supplementary Table 1.

### 2.3 Enzymatic Assay

Enzymatic inhibition assays were performed on LiTR as reported in (Ilari et al., 2018). Briefly, each assay was started by the addition of NADPH 100 μM to the pre-equilibrated reaction solution comprising HEPES 50 mM at pH 7.4, NaCl 40 mM, LiTR 50 nM, oxidized TSH 150 μM, and inhibitor. The decrease of absorbance at 340 nm, indicating the oxidation of NADPH, was measured to follow reaction progression.

The selected compounds were tested at a fixed concentration (100 μM, triplicates), and the residual activity with respect to control was considered.

### 2.4 Chemistry

All the commercially available reagents and solvents were purchased from Sigma-Aldrich, Alfa Aesar, VWR, and TCI and used without further purification. Reactions were followed by analytical thin-layer chromatography (TLC) on pre-coated TLC plates (layer: 0.20 mm silica gel 60 with a fluorescent indicator UV254, from Sigma-Aldrich). Developed plates were air-dried and analyzed under a UV lamp (UV 254/365 nm). A CEM Discover SP-focused microwave reactor was used for microwave-assisted reactions. Nuclear magnetic resonance (NMR) experiments were run on a Varian VXR 400 (400 MHz for ^1^H and 100 MHz for ^13^C). 1H and ^13^C NMR spectra were acquired at 300 K using deuterated chloroform (chloroform-*d*) and dimethyl sulfoxide (dimethyl sulfoxide-*d*_*6*_) as solvents. Chemical shifts (δ) are reported in parts per million (ppm) relative to tetramethylsilane (TMS) as the internal reference and coupling constants (*J*) are reported in hertz (Hz). The spin multiplicities are reported as s (singlet), br s (broad singlet), d (doublet), t (triplet), q (quartet), and m (multiplet). Mass spectra were recorded on a Waters ZQ4000, XevoG2-XSQTof, Acquity arc-QDA LC−MS apparatus with electrospray ionization (ESI) in positive mode. Compounds were named following IUPAC rules as applied by ChemBioDraw Ultra (version 19.0). The purity of compounds was determined using a Kinetex 5 μM EVO C18 100 Å, LC column 150 × 4.6 mm, and an HPLC JASCO Corporation (Tokyo, Japan) instrument (PU-1585 UV equipped with a 20 μl loop valve). All the tested compounds showed ≥95% purity by analytical HPLC.

#### 2.4.1 1-(4-(3-Phenylpropyl)piperazin-1-yl)ethan-1-one (1)

To a stirred solution of **8** (79 mg; 0.38 mmol, 1.0 eq) and triethylamine (0.07 ml, 0.48 mmol, 1.25 eq) in dichloromethane (4 ml) at 0°C, acetic anhydride (0.04 ml, 0.42 mmol, 1.1 eq) was added dropwise. The resulting reaction mixture was stirred at room temperature for 3 h. Upon completion, the reaction mixture was diluted with additional dichloromethane (10 ml) and washed with saturated NaHCO_3_ aqueous solution (2 × 10 ml). The organic phase was dried over anhydrous Na_2_SO_4_, filtered, and concentrated under *vacuum* to afford **1** as a colorless oil (86 mg, 92%). ^1^H NMR (400 MHz, chloroform-*d*) δ 7.32–7.24 (m, 3H), 7.23–7.15 (m, 2H), 3.62 (t, *J* = 5.2 Hz, 2H), 3.50–3.43 (m, 2H), 2.69–2.61 (m, 2H), 2.46–2.34 (m, 6H), 2.08 (s, 3H), 1.82 (p, *J* = 7.6 Hz, 2H). ^13^C NMR (101 MHz, chloroform-*d*) δ 169.0, 142.0, 128.5 (2C), 128.5 (2C), 126.0, 57.9 (2C), 53.4, 52.9, 46.5, 41.6, 33.7, 21.8.

#### 2.4.2 N-(4-fluorophenyl)-4-methylpiperazine-1-carboxamide (2)

To a solution of phenyl (4-fluorophenyl)carbamate (**13**, 84 mg, 0.36 mmol) in dichloromethane (1.85 ml) was added triethylamine (2.00 eq) and the methylpiperazine (**12**, 0.03 ml, 0.36 mmol). The reaction mixture was heated at 40°C overnight. Solvent was removed under *vacuum* and the resulting crude purified by silica gel chromatography. Purification by silica gel column chromatography (dichloromethane/methanol, 9.5:0.5), gave **2** as pale-yellow powder (54 mg, 64%).^1^H NMR (400 MHz, chloroform-*d*) δ 7.32–7.27 (m, 2H), 7.01–6.90 (m, 2H), 6.26 (br, 1H), 3.52–3.49 (m, 4H), 2.47–2.43 (m, 4H), 2.34 (s, 3H). ^13^C NMR (101 MHz, chloroform-*d*) δ 159.1 (d, *J*_*C-F*_ = 242.0 Hz), 155.2, 135.0 (d, *J*_*Cp-F*_ = 2.8 Hz), 122.2 (d, *J*_*Cm-F*_ = 8.0 Hz, 2C), 115.6 (d, *J*_*Co-F*_ = 22.4 Hz, 2C), 54.8, 46.3, 44.2.

#### 2.4.3 N-(4-fluorophenethyl)furan-2-carboxamide (3)

2-(4-Fluorophenyl)ethan-1-amine (**15**, 0.20 ml, 1.53 mmol) was added dropwise to a microwave vial charged with a magnetic stirring bar and the acyl chloride **14** (0.43 ml, 3.28 mmol) in toluene (3 ml). The reaction was carried out under microwave irradiation at 100°C, 150 W for 10 min. The solvent was removed under vacuum, and the crude purified by silica gel chromatography. Purification by silica gel column chromatography (dichloromethane/methanol, 9.8:0.2) gave **3** as yellowish oil (345 mg, 94%). ^1^H NMR (400 MHz, chloroform-*d*) δ 7.40 (dd, *J* = 1.8 and 0.8 Hz, 1H), 7.22–7.15 (m, 2H), 7.10 (dd, *J* = 3.6 and 0.8 Hz, 1H), 7.04–6.96 (m, 2H), 6.49 (dd, *J* = 3.6 and 1.8 Hz, 1H), 6.36 (s, 1H), 3.67–3.61 (m, 2H), 2.89 (t, *J* = 7.2 Hz, 2H). ^13^C NMR (101 MHz, CDCl_3_) δ 161.8 (d, *J *_*C-F*_ = 244.4 Hz), 158.5, 148.1, 143.9, 137.5, 134.5 (d, *J*_*Cp-F*_ = 3.2 Hz), 130.3 (d, *J*_*Cm-F*_ = 7.8 Hz, 2C), 115.6 (d, *J*_*Co-F*_ = 21.2 Hz, 2C), 114.3, 112.3, 40.5, 35.2, 21.7.

#### 2.4.4 Tert-Butyl Piperazine-1-carboxylate (5)

To a solution of piperazine (**4**, 4.04 g, 48.00 mmol, 6.0 eq) in dichloromethane (24 ml) at 0°C, was added a solution of Boc_2_O (1.75 g, 8.00 mmol, 1.0 eq) in dichloromethane (40 ml) over a period of 2 h. The reaction mixture was stirred at room temperature for 18 h. Upon completion, the resulting suspension was washed with H_2_O (5 × 100 ml) to remove the unreacted piperazine. The organic phase was dried over anhydrous Na_2_SO_4_, filtered, and concentrated under *vacuum* to afford **5** as a colorless solid (947 mg, 64%). ^1^H NMR (400 MHz, chloroform-*d*) δ 3.44–3.35 (m, 4H), 2.84–2.75 (m, 4H), 2.16 (br, 1H), 1.45 (s, 9H).

#### 2.4.5 Tert-Butyl 4-(3-Phenylpropyl)piperazine-1-carboxylate (7)

In a 50-ml sealed vessel, a stirred mixture of **5** (555 mg, 2.97 mmol, 1.5 eq) and commercially available (3-bromopropyl)benzene (**6**, 0.30 ml, 1.98 mmol, 1.0 eq) in acetonitrile (6 ml) in the presence of K_2_CO_3_ (411 mg; 2.97 mmol, 1.5 eq) as base and KI (3 mg; 0.02 mmol, 0.01 eq) as a catalyst, was heated to 80°C for 3 h. Upon completion, the hot suspension was filtered, and the residue was washed with acetone several times. The collected filtrates were concentrated under *vacuum* and the resultant crude was purified by silica gel column chromatography (dichloromethane/methanol/ammonia 32% aqueous solution, 9.8:0.2:0.02) to afford **7** as a colorless oil (554 mg, 92%). ^1^H NMR (400 MHz, chloroform-*d*) δ 7.31–7.23 (m, 2H), 7.22–7.14 (m, 3H), 3.44 (t, *J* = 5.2 Hz, 4H), 2.64 (t, *J* = 7.6 Hz, 2H), 2.40–2.35 (m, 6H), 1.84 (q, *J* = 7.6 Hz, 2H), 1.45 (s, 9H).

#### 2.4.6 1-(3-Phenylpropyl)piperazine (8)

**7** (368 mg, 1.21 mmol, 1.0 eq) was treated with trifluoroacetic acid (1.85 ml, 24.2 mmol, 20.0 eq) in dichloromethane (12 ml) at 0°C. The ice bath was removed, and the resulting mixture was stirred at room temperature for 2 h. Upon reaction completion, the mixture was diluted with additional dichloromethane (10 ml) and washed with saturated NaHCO_3_ aqueous solution (2 × 15 ml). The organic phase was dried over anhydrous Na_2_SO_4_, filtered, and concentrated under *vacuum* to afford **8** as a colorless oil (233 mg, 94%). ^1^H NMR (400 MHz, chloroform-*d*) δ 7.28–7.20 (m, 2H), 7.19–7.10 (m, 3H), 2.87 (t, *J* = 4.8 Hz, 4H), 2.65–2.56 (m, 2H), 2.46–2.27 (m, 6H), 2.20 (br, 1H), 1.85–1.73 (m, 2H).

#### 2.4.7 Phenyl (4-Fluorophenyl)carbamate (11)

To a solution of **9** (0.09 ml, 0.90 mmol, 1.0 eq) and Na_2_CO_3_ (57.2 mg, 0.54 mmol, 0.6 eq) in a mixture of ethyl acetate, tetrahydrofuran and H_2_O (1.36, 0.27 and 0.27 ml, respectively) at 0°C, phenyl chloroformate (**10,** 0.12 ml, 0.99 mmol, 1.1 eq) was added dropwise. The reaction mixture was stirred at room temperature overnight, then it was concentrated under a *vacuum* to remove organic solvents. Water was added to the residue and the resulting precipitate was recovered by filtration under *vacuum*, washed with water, and dried to give compound **11** as a grey solid (357 mg, quantitative yield). ^1^H NMR (400 MHz, chloroform-*d*) δ 7.44–7.37 (m, 4H), 7.26–7.22 (m, 1H), 7.21–7.17 (m, 2H), 7.08–7.01 (m, 2H), 6.88 (br, 1H).

#### 2.4.8 Furan-2-carbonyl Chloride (14)

SOCl_2_ (5.26 ml, 44.6 mmol) was added dropwise to a suspension of the furan-2-carboxylic acid (**13**, 500 mg, 4.46 mmol) in toluene (22 ml). The reaction refluxed at 110°C for 2 h before heating was stopped. Evaporation of the volatiles under *vacuum* gave the desired compound (assumed 100% yield), which was employed in the next synthetic step without further purification.

## 3 Results and Discussion

### 3.1 Fragment Screening: 12 Hits Identified in 5 Sites

Crystallographic fragment screening performed at the XChem facility allowed us to test 352 small compounds from the DSiP library, a collection specifically designed to ensure rapid and cheap follow-up synthesis (Cox et al., 2016).

**FIGURE 1:**
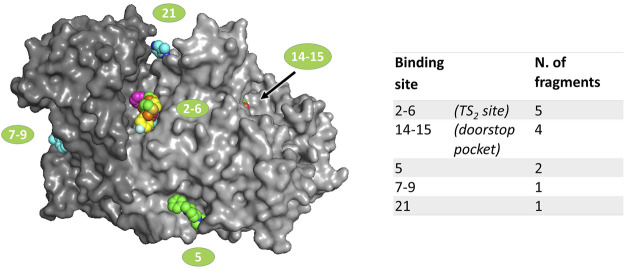
Location of fragments binding sites on TbTR surface. The two subunits of the dimer are colored in two different grey shades. Ligands are represented as spheres. Sites numbering is reported according to the output generated by PanDDA analysis; double numbers indicate symmetrically equivalent sites on the dimer.

Data analysis, performed by the PanDDA algorithm (Pearce et al., 2017), reported 480 interesting “events” clustered in 33 sites possibly associated to fragment binding. Visual inspection of these putative events led to the identification of 21 true binding events in eight sites, corresponding to 12 fragment hits distributed in 5 independent binding sites (Figure 1), described later in detail. The experimental results have been compared to the prediction made by FTmap (Kozakov et al., 2015), a computational mapping server that identifies binding hot spots (Supplementary Figure S3).

Table 1 gives an overview of data quality for the hits. All datasets have a resolution higher than 2 Å and the modeled fragments show a good correlation with the electron density maps, as proven by the real-space correlation coefficient (RSCC) (Pearce et al., 2017). Indeed, the RSCC is around 0.9 for most hits and always in the range of 0.77–0.93. Refined occupancy is relatively high for all the fragments apart from sample 90, but even in this case, the ligand has been unambiguously located thanks to the event map generated by PanDDA (see Supplementary Material for a view of the event map for each ligand).

**TABLE 1:** Overall data quality for fragment hits. R/Rf: R factor and free R factor as calculated by the last refinement cycle in Refmac. RSCC: real space correlation coefficient. The reference model is the structure of unbound TbTR based on the best dataset collected.

Sample	Pdb code	R/Rf	Resolution	Binding site	Occupancy	RSCC
60	5S9S	20.4/24.3	1.8	5	0.88	0.85
64	5S9T	18.9/22.1	1.66	14–15	0.7–0.64	0.93–0.77
68	5S9U	19.3/22.8	1.73	14–15	0.68–0.63	0.85–0.8
69	5S9V	18.7/22.9	1.9	2–6	1–0.96	0.83–0.87
71	5S9W	19.9/24.6	1.96	2–6	0.66–0.72	0.86–0.89
90	5S9X	19.7/23.8	1.84	5–14–15	0.29–0.5–0.63	0.87–0.89–0.85
94	5S9Y	19.8/23.6	1.75	9–7	0.86–0.7	0.9–0.84
109	5S9Z	19.3/22.5	1.73	6–2	0.8–0.92	0.86–0.91
117	5SA0	19.6/25.1	1.97	14–15	0.63-0.72	0.78-0.81
221	5SA1	19.4/23.1	1.84	2	1	0.93
296	5SA2	20/23.8	1.78	21	0.8	0.84
371	5SA3	18.7/21.8	1.74	6	0.76	0.87
Reference model	5SMJ	19.3/21.8	1.65	-	-	-

### 3.2 Fragments Binding in Proximity of the NADPH-Site: The Doorstop-Pocket

**FIGURE 2:**
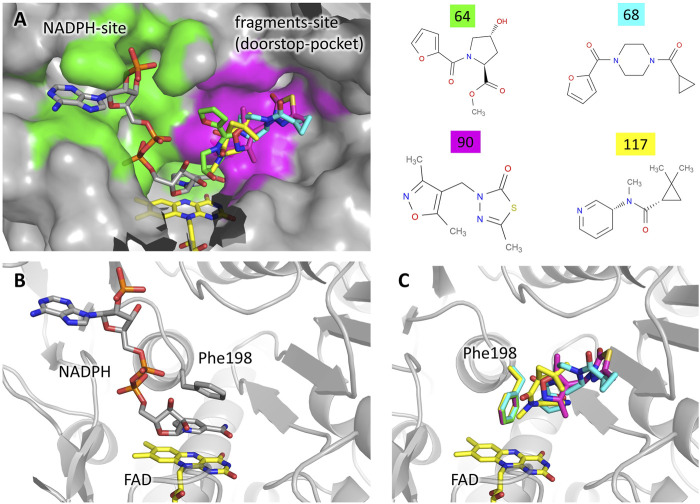
Fragments binding to the “doorstop pocket”. **(A)** View of the NADPH binding site (green) and the fragment binding site (magenta). Although soaking has been carried out in absence of NADPH, it is shown in the figure for clarity. The figure was prepared by adding the fragments (color code on the left) to the structure of the complex TbTR-NADPH (pdb: 2wov). **(B)** Conformation of Phe198 upon NADPH binding. **(C)** Conformation of Phe198 in presence of the fragments, hampering NADPH binding.

Four different fragments bind to the site 14–15. The binding does not cause any significant structural variation with respect to the unbound protein. A detailed description of the interactions engaged by each fragment is reported in Supplementary Figure S1. The analysis of the unbound TbTR structure made by FTmap predicts a hot spot located at this site, although it does not stand out as a remarkable druggable site (Supplementary Figure S3). Site 14–15 is located in close proximity to the NADPH binding cavity (Figure 2). As shown in Figure 2A, the two binding pockets are almost independent (note that fragment soaking has been performed in the absence of NADPH). Although there is no significant overlap between the fragments and NADPH (Figure 2A), the presence of the fragments would hamper the entrance of NADPH into its cavity due to the conformation assumed by Phe198 (Figures 2B,C). In fact, Phe198 is located over the FAD and, in the apo conformation, its side chain is perpendicular to the isoalloxazine ring. Upon NADPH binding, the nicotinamide moiety inserts between FAD and Phe198 forming an aromatic sandwich in which the phenyl ring lays parallel to the FAD (Figure 2B). Therefore, for the NADPH to bind to TbTR, it is essential that Phe198 is able to switch from its perpendicular position to a parallel one with respect to the FAD. The binding of the fragments in the adjacent pocket blocks Phe198 in the perpendicular conformation, thus the binding of the cofactor is prevented (Figure 2C).

A very similar mechanism of inhibition has been observed in 2018 for the protein thioredoxin glutathione reductase from *Schistosoma mansoni* (SmTGR) by Angelucci and coworkers (Silvestri et al., 2018). The authors defined the fragment binding site as the “doorstop pocket”, according to the effect to block the aromatic side chain that acts as a door for the entrance of NADPH. Indeed, the mode of binding of NADPH described above for TR from *Trypanosoma brucei* is common to NAD/FAD reductases in general, only differing by the nature of the “door” residue that may either be a phenylalanine or a tyrosine residue depending on the species.

By sequence comparison with homologous, the authors noted that the residues contributing to the doorstop pocket in SmTGR are highly conserved, and they predicted the existence of a similar pocket in TR from leishmania and trypanosoma as well as in human GR and thioredoxin reductase (TrxR).

**FIGURE 3:**
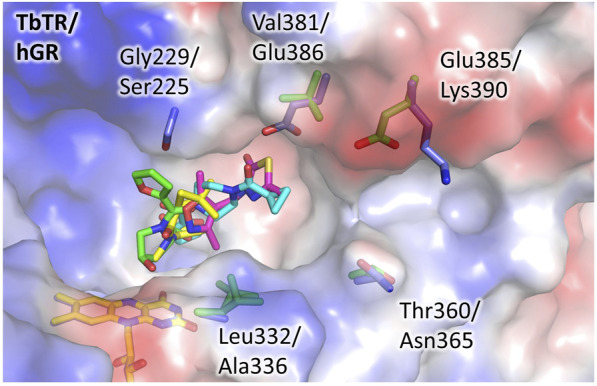
The doorstop pocket, differences between TbTR and hGR. The electrostatic semitransparent surface of TbTR in complex with the fragment 64 (pdb: 5S9T) is reported as generated by PyMol. Yellow arrows indicate the main sequence substitutions, the corresponding side chains are shown as sticks (green for TbTR, blue for hGR (pdb: 3djj)).

To evaluate the feasibility to exploit this pocket to develop selective inhibitors of TbTR, we compared its structure to hGR and detected some significant differences (Figure 3). We found few substitutions among the residues lining the pocket, namely Gly229 and Leu332 replaced by Ser225 and Ala336 in hGR, that cause a shape variation that is limited but capable to interfere with the binding. However, more relevant substitutions are present not far away from the pocket, and concern residues Val381, Glu385, and Thr360 which are replaced by Glu386, Lys390, and Asn365 in hGR. These substitutions, affecting both shape and charge distribution, are located along a small contiguous cleft (Figure 3) that could be targeted by fragment growing. Notably, other human NAD-dependent reductases, such as TRxR, should be taken into account as potential off-targets.

### 3.3 Fragments Binding to the TSH Cavity

**FIGURE 4:**
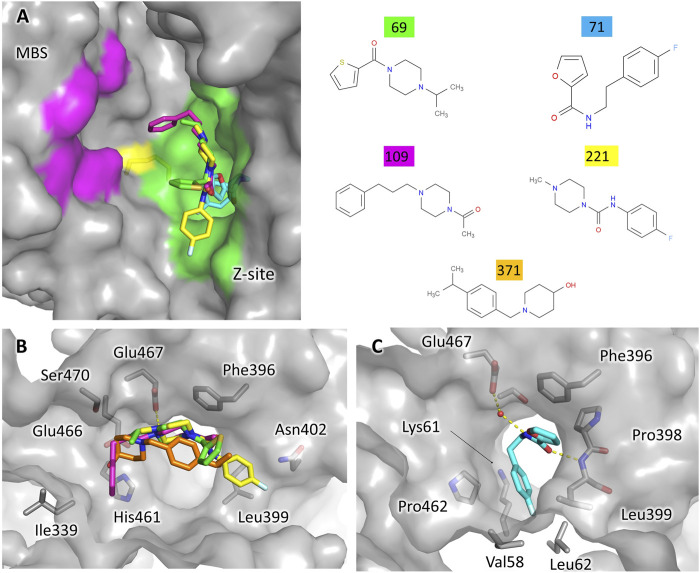
Fragments binding at the TSH cavity. **(A)** View of the whole TSH cavity. The surfaces corresponding to the Z-site and the MBS are colored green and magenta, respectively. The redox active cysteines are shown as sticks and colored yellow to help locate the fragments with respect to the catalytic site. **(B)** Binding of the fragments 69, 109, 221, and 371 are characterized by an amino heterocycle. **(C)** Binding of the fragment 71.

Five fragments were found to bind at the site two to six, located in the TSH cavity. The ligands occupy the same region of the cavity, corresponding to the so-called Z-site, a sub-pocket predicted by bioinformatic analysis (Khan et al., 2000) but never confirmed experimentally until now (Figure 4).

Surprisingly, no ligands could be modeled at the mepacrine binding site (MBS), although most of the yet structurally characterized inhibitors of TR are known to target this hot-spot (Battista et al., 2020), including the ones mispredicted to bind the Z-site (Saravanamuthu et al., 2004). Indeed, the PanDDA analysis reported some events at the MBS, but the density map was so poorly defined that these putative hits were not further considered. This result is confirmed by FTmap since no hot spots are predicted at the MBS (Supplementary Figure S3).

Some relevant features can be detected among the fragments targeting the Z-site. Four fragments contain an amino heterocycle, namely a piperazine ring for hits 69, 109, 221 or a piperidine ring for hits 371 (Figure 4B). It is worth noticing that the piperazine assumes the same position in all the structures. In general, the amino heterocycle strongly contributes to binding, in fact, the positively charged amino group forms a salt bridge with Glu467 (N-O distance: 2.7 Å for piperazine, 3.6 Å for piperidine), while the other main interactions are hydrophobic and involve Ile339, Phe396, Leu399 (Figure 4B).

The fifth fragment, compound 71, strongly differs in the mode of binding: it explores a small cavity corresponding to the narrow entrance of a wide interfacial cavity that connects the two TSH sites on the dimer (Figure 4C). The interaction is mainly hydrophobic: the fluorophenyl group targets a sub-pocket lined mostly by hydrophobic side chains (Figure 4C) while the furan makes a stacking interaction with Phe396. Moreover, the amide moiety engages in hydrogen bonds with the Leu399 backbone and a water molecule bridging to Glu367.

The binding of the ligands to the Z-site does not require any conformational rearrangement of the site with respect to the apo structure (ground state model, PDB ID 5SMJ), except a very limited movement of the stretch 395–397 toward the ligand in the case of fragment 71.

Although no known inhibitor binds this area of the cavity, a HEPES molecule was serendipitously found in the TbTR structure solved in a complex with an inhibitor targeting the MBS (De Gasparo et al., 2019). It is interesting to note that the piperazine of HEPES interacts with Glu467 while the sulfonate points toward the subpocket. This finding stresses a role for the piperazine as an anchoring moiety for the Z-site.

Intriguingly, the interfacial cavity, whose entrance appears to be occluded by hit 71, has been exploited to carry out an alternative inhibition strategy of TR aimed at the disruption of the dimeric assembly (Ruiz-Santaquiteria et al., 2017; Revuelto et al., 2021). Moreover, a similar cavity is present in GR and is targeted by some non-competitive or uncompetitive inhibitors such as xanthenes (Savvides and Karplus, 1996), menadione (Karplus et al., 1989), and N-arylisoalloxazines (Schönleben-Janas et al., 1996). Indeed, the analysis made using the FTMap web server (Supplementary Figure S3) highlights hot-spots corresponding to hit 71 and indicates the whole interfacial cavity as a significant druggable site. The fact that no hits have been found within the cavity could be due to poor accessibility, resulting from the combination of the narrow entrance and the structural rigidity caused by crystal packing.

**FIGURE 5:**
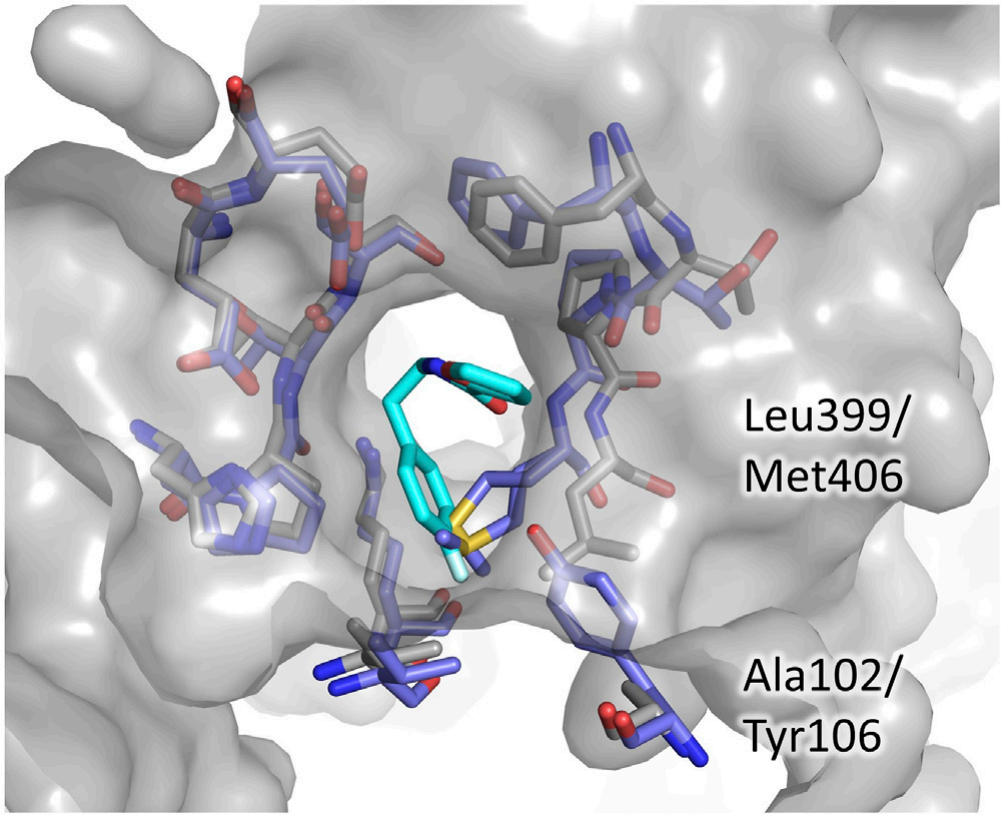
The Z-site, differences between TbTR and hGR. The residues lining the Z-site are shown as sticks, grey for TbTR, blue for hGR, and fragment 71 are colored cyan. Labels are reported only for residues differing in TR and GR.

Even in the case of the Z-site, we assessed the potentiality for selective inhibition by comparison to hGR. As shown in Figure 5, almost all the residues contributing to fragment binding are conserved apart for Leu399 which is replaced by Met406. This finding is not surprising because, during catalysis, this zone of the cavity hosts the carboxylic ends shared by both TSH and glutathione. However, structure superimposition shows that in hGR the small cavity targeted by fragment 71 is narrower due to the shift of the segment 396–399 (403–406 in hGR). The difference in conformation is justified by the replacement of Ala102 with the bulkier Tyr106: the side chain, therefore, pushes Met406 and results in a shrunk subpocket where the entrance of the ligand would be hampered (Figure 5).

This observation indicates that, in order to develop a selective inhibitor targeting the Z-site, it is essential to exploit this small cavity explored by fragment 71.

### 3.4 Other Binding Sites

The remaining binding sites (5, 7–9, 21) are less populated by fragments and are located in regions that seem not particularly significant for the modulation of the activity of the protein. Thus, these sites are likely of little relevance and the corresponding fragments will be only briefly described.

The binding sites of the fragments 60 and 90 are very close, thus they are grouped as site 5 even if they are distinct. In fact, while compound 60 binds at a mainly hydrophobic pocket on the protein surface, compound 90 is completely buried within the dimerization interface (Supplementary Figure S2). Note that compound 90 mainly binds to sites 14–15 (the doorstop pocket) while its occupancy in site 5 is low. Site 5 is far away from the catalytic site, and the compound binding does not cause any significant conformational variation, except the shift of the side chains of Leu73 and Phe83 in the case of hit 90 (Supplementary Figure S2B), suggesting that the binding of these ligands may not affect TbTR activity. However, it is worth noticing that fragment 90 in site 5 is adjacent to the interfacial cavity where the xanthene inhibitor of GR is bound (Supplementary Figure S3).

Fragments 94 and 296 are placed at sites 7–9 and 21 that are rather peripheral and solvent exposed.

### 3.5 Synthesis of Selected Fragments Targeting the Z-Site

Based on the structure analysis, we decided to focus on the fragments binding to the Z-site. Indeed, these hits offer the opportunity to improve already known inhibitors as well as to design completely new compounds by merging and linking approaches. We selected three representative fragments, i.e., two fragments containing the piperazine ring (109 and 221) and the one targeting the sub-pocket (71).

**SCHEME 1:**
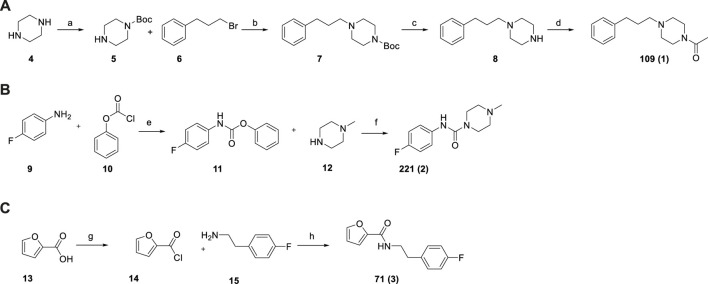
Synthesis of fragments 109 **(A)**, 221 **(B)**, 71 **(C)**. Reagent and conditions: **(a)** Boc_2_O, DCM, r.t., overnight (64%); **(b)** K_2_CO_3_, KI, CH_3_CN, 80°C, 3 h (92%); **(c)** TFA, DCM, from 0°C to r.t., 2 h (94%); **(d)** acetic anhydride, DCM, Et_3_N, r.t., 3 h (92%); **(e)** Na_2_CO_3_, THF/EA/H2O, r.t., 12 h (100%) **(f)** DCM, Et_3_N, 40°C, overnight (64%); **(g)** SOCl_2_, toluene, 110°C, 2 h (100% assumed); **(h) **toluene, MW irradiation, 100°C, 10 min, 150 W (94%).

Given the in-house availability of the building blocks, we decided to synthesize the compounds **109 *(1),* 221 *(2),*** and **71 *(3)***, over purchasing them from commercial vendors. Fragments **1**–**3** were thus synthesized by slightly modified reported procedures (Scheme 1) (Glennon et al., 1981; McNaughton et al., 2009; Li et al., 2019). In this way, the optimized synthetic routes also give clues about the synthetic feasibility, and the scale-up and provide the basis for analog expansion. All the synthesized compounds showed ≥95% purity by analytical HPLC and were characterized by spectroscopic data (^1^H and ^13^C NMR), which are reported in the *Methods* Section.

### 3.6 Activity Assay of Selected Fragments

We tested the activity of the selected fragments by preliminary inhibition assay on TR from *Leishmania infantum*. Indeed, the high degree of sequence conservation, reaching 100% for the TSH cavity, allows using TR from any species.

**FIGURE 6:**
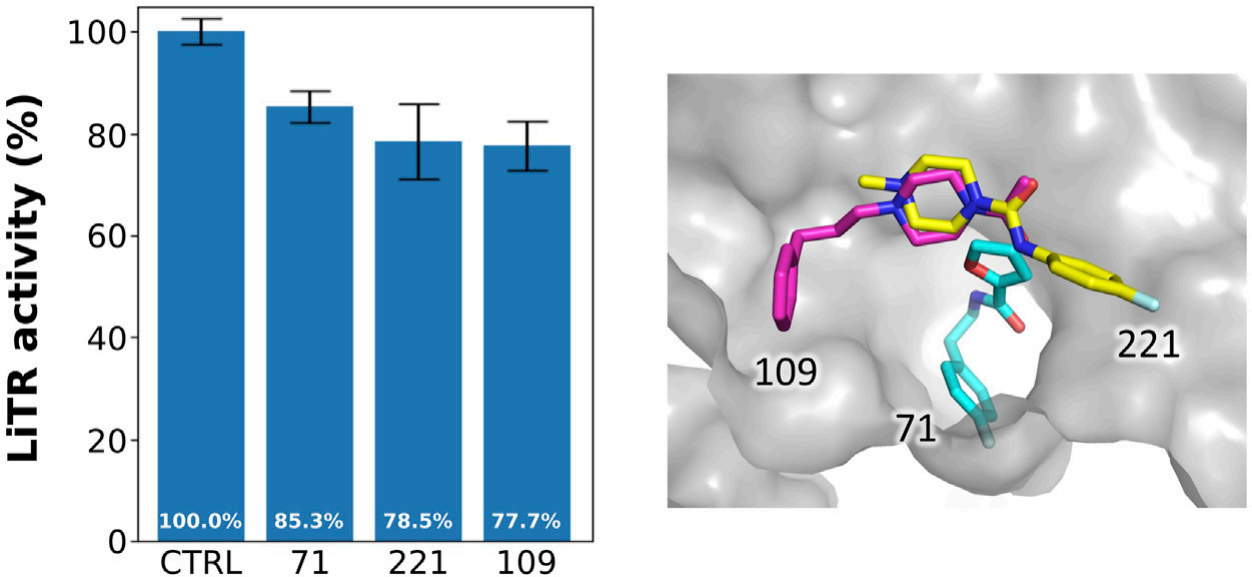
Activity of the selected fragments. Residual activity of LiTR after treatment with fixed concentration (100 μM) of the selected fragments.

As shown in Figure 6, reporting the residual activity of LiTR after treatment with a fixed concentration (100 μM) of the compounds, the fragments have a low but evident effect on protein activity, in line with the general behavior of fragments, whose affinity for the target is usually in the millimolar range due to the small size of these compounds that limits the number of possible interactions.

## 4 Conclusion and Perspectives

TR is an appealing but challenging target for the development of new trypanocidal drugs. Indeed, due to its high efficiency and turnover, only very potent inhibitors can be considered as leads, but the structural characteristics of the substrate binding cavity, wide and solvent exposed, make this goal difficult to achieve.

In order to bring out new opportunities for inhibition, such as new binding molecular scaffolds as well as uncharacterized secondary sites, we performed the first crystallographic fragment screening ever reported on TR. The experiment resulted in 12 fragments targeting 5 independent sites, two of which are of particular interest.

The screening revealed the existence of an allosteric pocket close to the NADPH binding site, named “doorstop pocket” in reference to the mechanism of inhibition observed. A very similar site has been recently detected in a homolog protein from the parasite Schistosoma and was successfully exploited to develop efficient inhibitors active in the low micromolar range (Silvestri et al., 2018), stressing the potentiality of the new druggable site identified by our study.

The second site, known as the Z-site, is located within the large TSH cavity but corresponds to a region not yet exploited for inhibition. The fragments binding to this site have some remarkable features making them ideal for follow-up optimization. Indeed, the recurrence of a piperazine moiety in three out of five fragments suggests a convenient anchoring point, suitable for fragment merging. Moreover, the capability of one fragment to target a small and specific subpocket provides an opportunity for specific inhibition. Based on these observations, we selected and synthesized three hits particularly suited for future developments and tested their activity on TR resulting to be in line with inhibitors with such a limited size. We are currently working on the design and synthesis of derivatives of the selected fragments based on the merging and linking approach. Moreover, the fact that most structurally characterized inhibitors bind to other regions of the same cavity suggests the possibility to exploit our results to modify known inhibitors to improve their activity and selectivity, possibly overcoming the constitutive limitations of TR as a drug target.

The promising results gathered from this first fragment screening campaign bring new light to TR as a target for a structure-based approach and encourage the expansion of the chemical space explored by testing new fragments collections.

## Data Availability

The datasets presented in this study can be found in online repositories. The names of the repository/repositories and accession number(s) can be found at: http://www.wwpdb.org/, 5S9S, 5S9T, 5S9U, 5S9V, 5S9W, 5S9X, 5S9Y, 5S9Z, 5SA0, 5SA1, 5SA2, 5SA3, and 5SMJ.
