# Supplementary material for: Innovative Approach for a Classic Target: Fragment Screening on Trypanothione Reductase Reveals New Opportunities for Drug Design
Source: Front Mol Biosci. 2022 Jul 4;9:900882. doi: 10.3389/fmolb.2022.900882 (PMC9289546; doi:10.3389/fmolb.2022.900882)
Supplement: Supplementary file 1 [file DataSheet1.pdf]

SUPPLEMENTARY MATERIAL

Table S1. X-ray data reduction and refinement statistics

Data are extracted from XCE processing. Data indicated by * are taken from the report generated upon PDB submission. Data in parentheses refer to high resolution shell.

**Sample**	**60**	**64**	**68**	**69**
**Pdb Id**	5S9S	5S9T	5S9U	5S9V
**Compound code**	Z2856434898	Z1614545742	Z32327641	Z2856434826
**Resolution Range**	77.95 - 1.80 (1.84 - 1.80)	78.10 - 1.66 (1.70 - 1.66)	65.10 - 1.73 (1.77 - 1.73)	65.19 - 1.90 (1.95 - 1.90)
**Wavelength**	0.9126	0.9126	0.9126	0.9126
**Space Group**	P 21 21 21	P 21 21 21	P 21 21 21	P 21 21 21
**Cell (a b c)**	80.28 108.60 111.95	80.35 108.90 112.07	79.96 108.51 111.68	80.03 108.79 111.93
**Cell (α β γ)**	90.00 90.00 90.00	90.00 90.00 90.00	90.00 90.00 90.00	90.00 90.00 90.00
**Total Reflections**	608303 (90225)	763136 (51913)	679474 (51498)	518009 (38074)
**Unique Reflections**	91672 (13227)	116537 (8546)	101875 (7461)	77649 (5699)
**Multiplicity**	6.60 (6.80)	6.50 (6.10)	6.70 (6.90)	6.70 (6.70)
**Completeness (%)**	100.00 (100.00)	100.00 (100.00)	100.00 (100.00)	100.00 (99.90)
**Mean I/Sigma(I)**	8.20 (0.9)	10.20 (0.8)	13.00 (0.9)	11.20 (0.8)
**R-Merge**	0.133 (2.100)	0.108 (2.192)	0.077 (2.173)	0.095 (2.285)
**R-Rim**	0.056 (0.863)	0.046 (0.964)	0.032 (0.888)	0.040 (0.949)
**Cc-Half**	0.997 (0.409)	0.997 (0.312)	0.999 (0.337)	0.999 (0.330)
**R-Factor**	0.202 (0.371)	0.188 (0.368)	0.192 (0.349)	0.184 (0.354)
**R-Free**	0.244 (0.393)	0.221 (0.378)	0.229 (0.353)	0.229 (0.324)
**N. of total atoms**	8130	8140	8151	8153
**Atoms for ligands**	171	183	185	185
**Atoms for waters**	518	524	525	527
**N. of polymer residues**	969	969	969	969
**Wilson B-Factor (Å)***	28.9	26.1	29.2	36.7
**Average B-Factor**	33.8	29.5	33.8	41.4
**B-Factor for ligands**	41.7	35.8	40.4	52.1
**B-Factor for solvent**	37.2	33.8	38.0	45.5
**Rms(Bonds)**	0.008	0.010	0.009	0.008
**Rms(Angles)**	1.507	1.592	1.556	1.485
**Ramachandran Favored (%)***	96.5	98	97.5	96.5

**Ramachandran Allowed (%)***	3.5	2	2.5	3.5

**Sample**	**71**	**90**	**94**	**109**
**Pdb Id**	5S9W	5S9X	5S9Y	5S9Z
**Compound code**	Z26769872	Z1899842917	Z24758179	Z2856434884
**Resolution Range**	78.14 - 1.96 (2.01 - 1.96)	78.25 - 1.84 (1.89 - 1.84)	64.33 - 1.75 (1.79 - 1.75)	65.07 - 1.73 (1.77 - 1.73)
**Wavelength**	0.9126	0.9126	0.9126	0.9126
**Space Group**	P 21 21 21	P 21 21 21	P 21 21 21	P 21 21 21
**Cell (a b c)**	79.65 109.11 111.97	79.98 109.24 112.13	79.77 108.36 112.07	79.78 108.47 112.04
**Cell (α β γ)**	90.00 90.00 90.00	90.00 90.00 90.00	90.00 90.00 90.00	90.00 90.00 90.00
**Total Reflections**	406778 (23511)	571871 (42736)	662683 (98477)	693026 (103167)
**Unique Reflections**	70595 (5151)	85835 (6279)	98469 (14212)	102308 (14712)
**Multiplicity**	5.80 (4.60)	6.70 (6.80)	6.70 (6.90)	6.80 (7.00)
**Completeness (%)**	99.90 (99.80)	100.00 (100.00)	100.00 (100.00)	99.80 (99.40)
**Mean I/Sigma(I)**	9.40 (0.8)	10.80 (0.8)	13.60 (0.9)	14.00 (1.0)
**R-Merge**	0.100 (1.944)	0.099 (2.552)	0.068 (1.937)	0.060 (1.853)
**R-Rim**	0.044 (1.002)	0.042 (1.054)	0.028 (0.793)	0.025 (0.745)
**Cc-Half**	0.998 (0.323)	0.998 (0.297)	0.999 (0.434)	0.999 (0.490)
**R-Factor**	0.196 (0.384)	0.195 (0.365)	0.196 (0.356)	0.191 (0.339)
**R-Free**	0.247 (0.386)	0.238 (0.364)	0.236 (0.350)	0.227 (0.342)
**N. of total atoms**	8148	8152	8149	8156
**Atoms for ligands**	187	186	183	189
**Atoms for waters**	520	525	525	526
**N. of polymer residues**	969	969	969	969
**Wilson B-Factor (Å)***	39.3	34.6	33.4	34.2
**Average B-Factor**	47.2	39.0	38.7	38.1
**B-Factor for ligands**	54.6	44.5	45.4	44.3
**B-Factor for solvent**	51.3	42.9	43.0	43.6
**Rms(Bonds)**	0.009	0.008	0.008	0.009
**Rms(Angles)**	1.459	1.483	1.514	1.543
**Ramachandran Favored (%)***	96	96	96	97
**Ramachandran Allowed (%)***	4	4	3	3

Sample	117	221	296	371
Pdb Id	5SA0	5SA1	5SA2	5SA3
Compound code	Z1506050651	Z2856434944	Z1148747945	Z2856434874
Resolution Range	77.97 - 1.97 (2.02 - 1.97)	56.09 - 1.84 (1.89 - 1.84)	78.03 - 1.78 (1.83 - 1.78)	77.99 - 1.74 (1.78 - 1.74)
Wavelength	0.9126	0.9126	0.9126	0.9127
Space Group	P 21 21 21	P 21 21 21	P 21 21 21	P 21 21 21
Cell (a b c)	79.58 108.28 112.36	80.06 109.06 112.05	80.05 108.70 112.09	80.57 108.62 112.04
Cell (α β γ)	90.00 90.00 90.00	90.00 90.00 90.00	90.00 90.00 90.00	90.00 90.00 90.00
Total Reflections	463305 (63701)	571346 (42442)	632270 (47782)	680647 (51332)
Unique Reflections	69798 (10045)	85702 (6271)	94232 (6873)	100864 (7344)
Multiplicity	6.60 (6.30)	6.70 (6.80)	6.70 (7.00)	6.70 (7.00)
Completeness (%)	100.00 (100.00)	100.00 (100.00)	100.00 (100.00)	99.60 (99.00)
Mean I/Sigma(I)	9.40 (0.8)	10.00 (0.8)	10.30 (0.7)	11.30 (0.9)
R-Merge	0.105 (1.968)	0.105 (2.393)	0.093 (2.620)	0.085 (2.216)
R-Rim	0.044 (0.843)	0.044 (0.989)	0.039 (1.062)	0.035 (0.894)
Cc-Half	0.998 (0.421)	0.998 (0.302)	0.999 (0.343)	0.998 (0.324)
R-Factor	0.193 (0.352)	0.192 (0.350)	0.198 (0.371)	0.185 (0.329)
R-Free	0.251 (0.387)	0.232 (0.366)	0.238 (0.388)	0.217 (0.312)
N. of total atoms	8119	8149	8122	8145
Atoms for ligands	179	170	164	170
Atoms for waters	520	538	529	534
N. of polymer residues	968	969	969	969
Wilson B-Factor (Å)*	41.3	32.5	33.3	30.1
Average B-Factor	47.9	36.8	35.8	33.7
B-Factor for ligands	51.9	45.1	42.5	40.7
B-Factor for solvent	52.0	40.1	40.3	39.6
Rms(Bonds)	0.009	0.009	0.009	0.009
Rms(Angles)	1.651	1.518	1.541	1.544
Ramachandran Favored (%)*	95	97	97	98
Ramachandran Allowed (%)*	4	3	3	2

Table S2. Structures, accession codes, and electron density maps of fragment hits.

Sample	PDB ID	Compound (SMILES, ID)	PanDDA maps (Z-map: red/green, σ = ±3 Event map: blue, σ = 2)	2Fo-Fc map (1σ)
**60**	5S9S	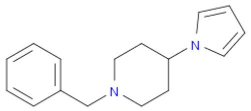 C(N1CCC(CC1)N2C=CC=C2)C=3C=CC=CC3 Z2856434898	Site 5 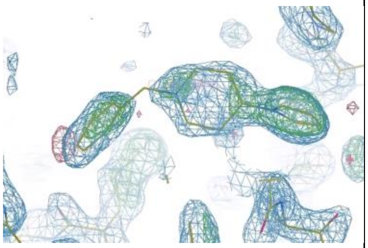 1-BDC = 0.22	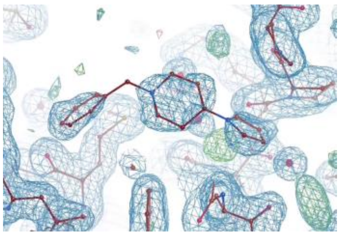
**64**	5S9T	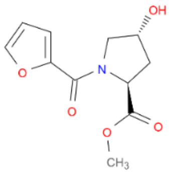 COC(=O)[C@@H]1C[C@@H](O)CN1C(=O)C2=CC=CO2 Z1614545742	Site 14 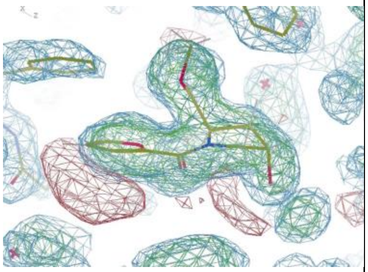 1- BDC = 0.18	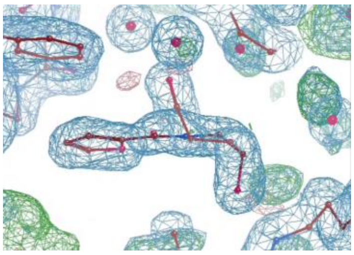
**68**	5S9U	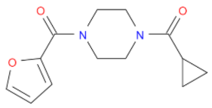 O=C(C1CC1)N2CCN(CC2)C(=O)C3=CC=CO3 Z32327641	Site 14 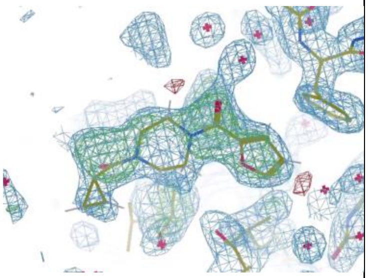 1-BDC = 0.14	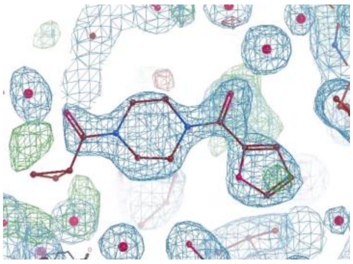
**69**	5S9V	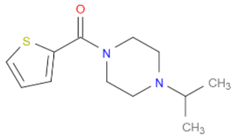 CC(C)N1CCN(CC1)C(=O)C2=CC=CS2 Z2856434826	Site 6 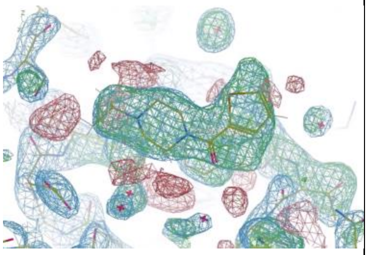 1-BDC = 0.25	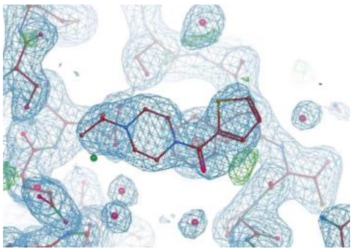

**71**	5S9W	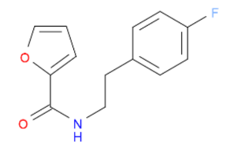 FC=1C=CC(CCNC(=O)C2=CC=CO2)=CC1 Z26769872	Site 6 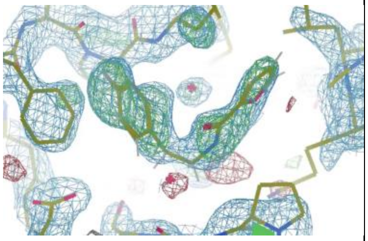 1-BDC = 0.27	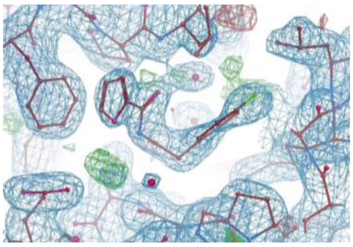	
**90**	5S9X	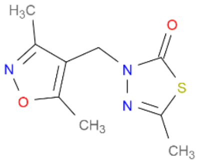 CC1=NN(CC=2C(C)=NOC2C)C(=O)S1 Z1899842917	Site 5 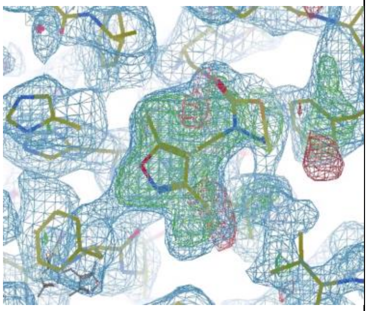 1-BDC = 0.09	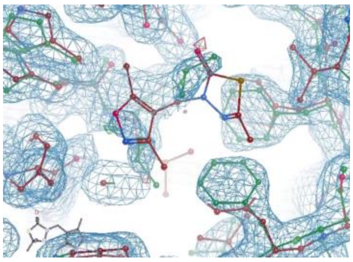 (green model = ground state)	
			Site 15 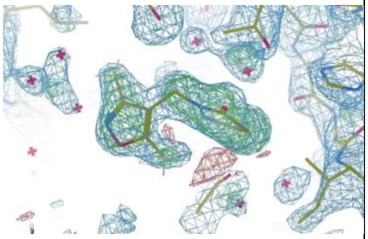 1-BDC = 0.14	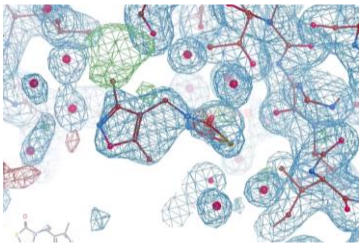	
**94**	5S9Y	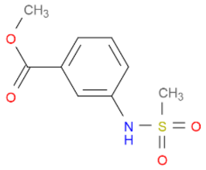 COC(=O)C=1C=CC=C(NS(=O)(=O)C)C1 Z24758179	Site 9 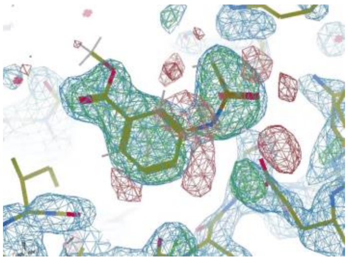 1-BDC = 0.19	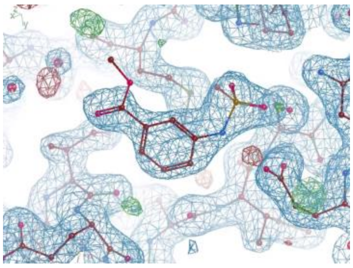	
**109**	5S9Z	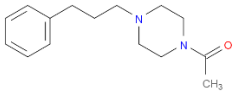 CC(=O)N1CCN(CCCC=2C=CC=CC2)CC1 Z2856434884	Site 6 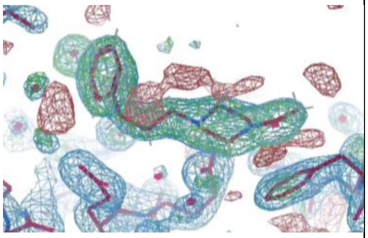 1-BDC = 0.25	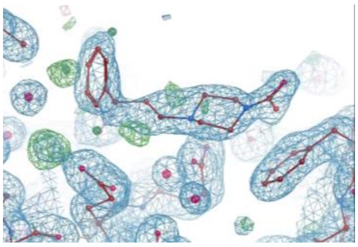	

**117**	5SA0	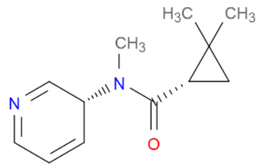<br>CN(C(=O)[C@H]1CC1(C)C)C=2C=CC=NC2<br>Z1506050651	Site 14<br>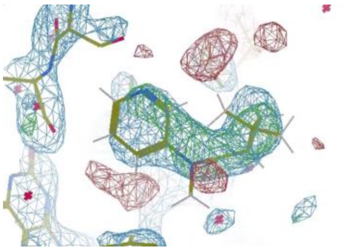<br>1-BDC = 0.29	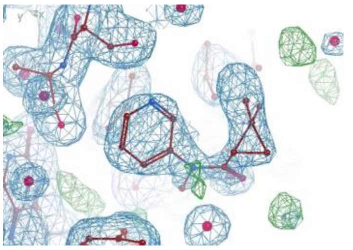
**221**	5SA1	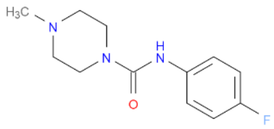<br>CN1CCN(CC1)C(=O)NC=2C=CC(F)=CC2<br>Z2856434944	Site 2<br>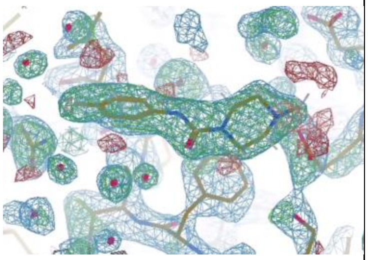<br>1-BDC = 0.25	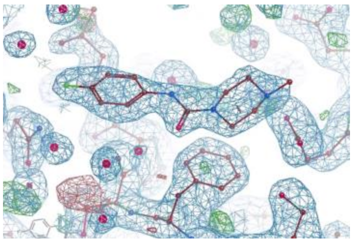
**296**	5SA2	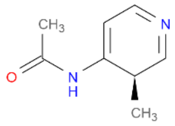<br>CC(=O)NC=1C=CN=CC1C<br>Z1148747945	Site 21<br>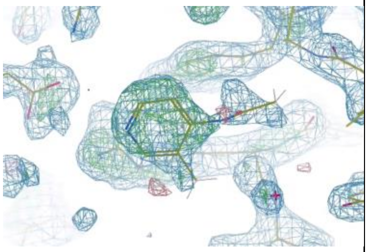<br>1-BDC = 0.20	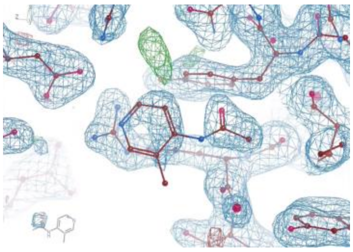
**371**	5SA3	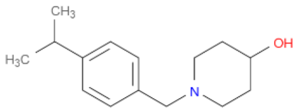<br>CC(C)C=1C=CC(CN2CCC(O)CC2)=CC1<br>Z2856434874	Site 6<br>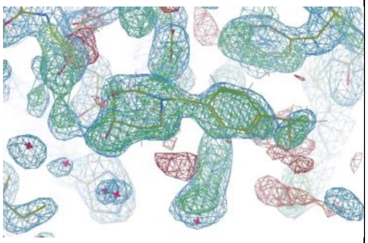<br>1-BDC = 0.24	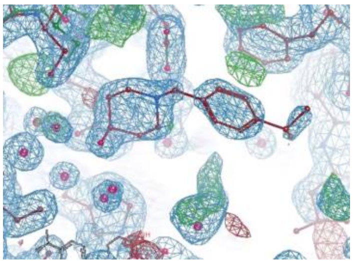

Figure S1. Fragments binding at the doorstop pocket.

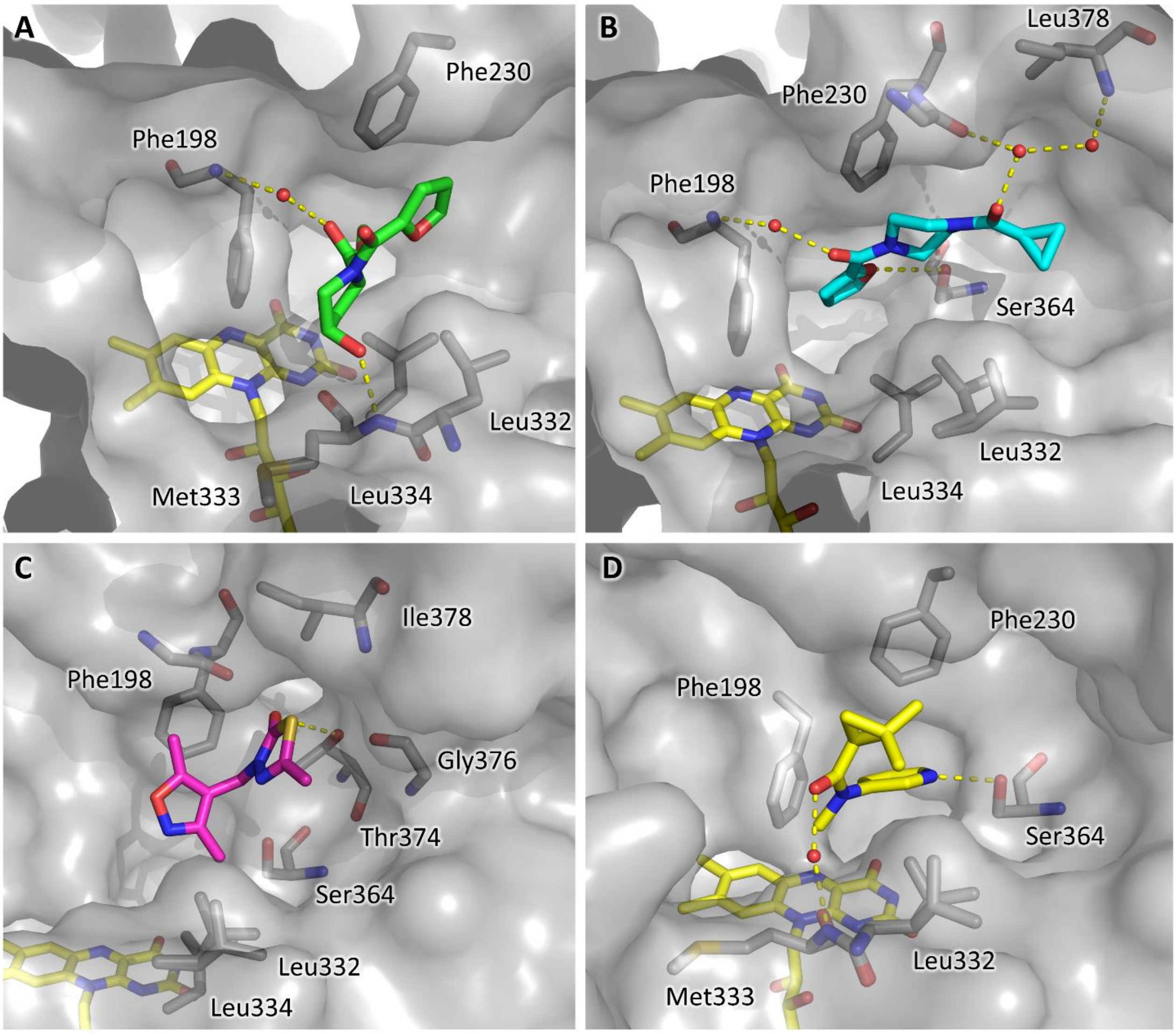

Fragments 64 (A, green), 68 (B, cyan), 90 (C, magenta) and 117 (D, yellow). FAD is shown as yellow sticks. Residues mainly involved in the interaction are indicated. Water molecules are represented as red spheres. Polar interactions are represented as dashed lines.

Figure S2. Fragments binding to less relevant sites.

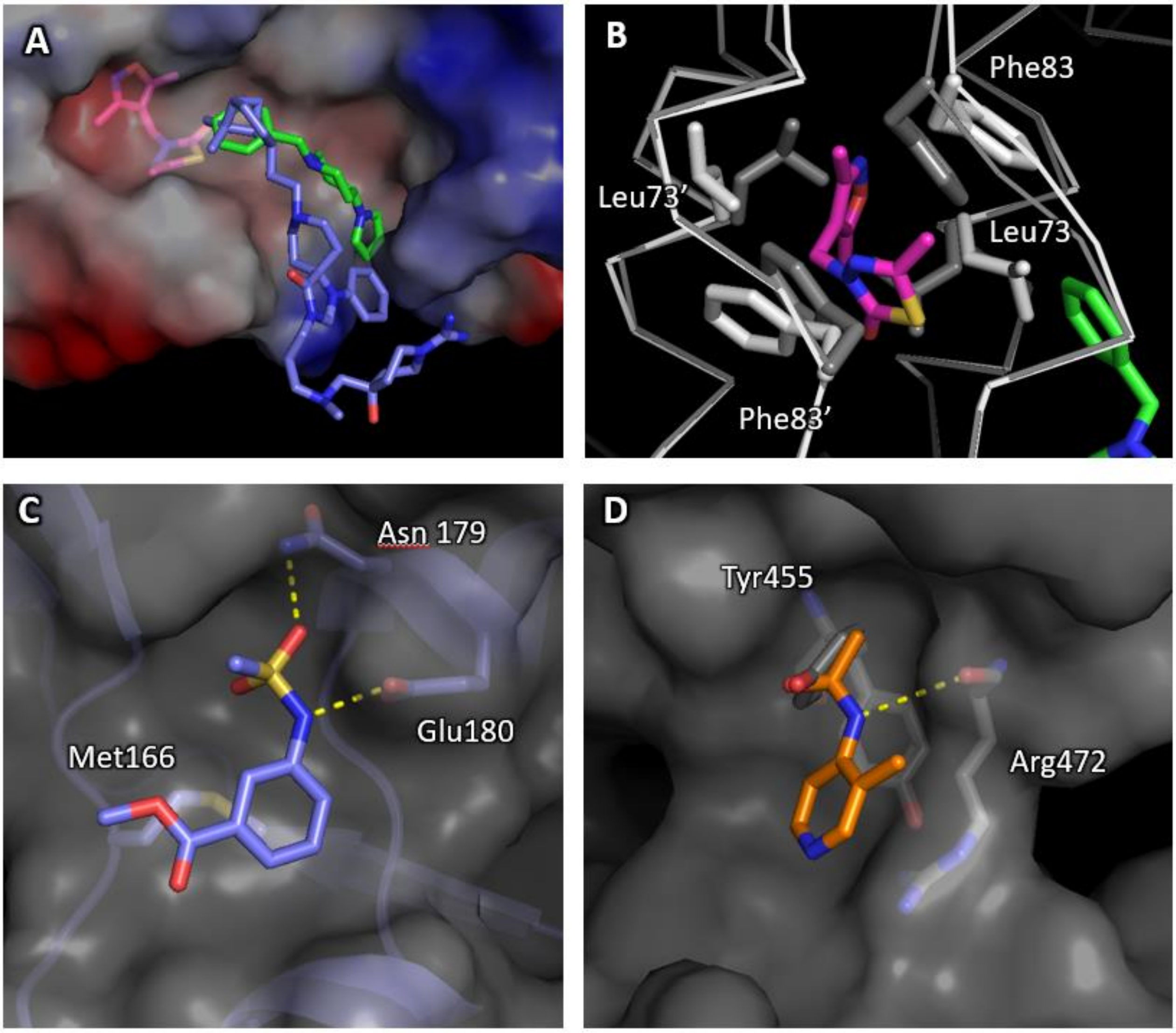

(A) Fragment 60 is shown in green. A previously characterized inhibitor (pdb 6BR5), found to bind at the TS_2 cavity and at this site, is shown in blue. Note fragment 90 in pink, buried within the protein and visible through the transparent surface. (B) Fragment 90, colored pink, is buried in the dimerization interface. The apo (dark grey) and fragment bound (light grey) structures are represented as ribbon and sticks, showing that the binding requires the shift of the side chains of Leu73 and Phe83 of each subunit. (C) Binding of fragment 94 (blue). (D) Binding of fragment 296 (orange). The side chain of Tyr455 displays two conformations in the ligand-free TbTR (ground-state, dark grey), the buried one is selected upon fragment 296 binding. Polar interactions are indicated as dashed lines.

Figure S3. FTmap analysis and comparison with experimental fragment binding.

We analyzed the structure of unbound TbTR (ground-state model, PDB ID 5SMJ) by FTmap, a computational mapping server that identifies binding hot spots of macromolecules (Kozakov et al, 2015) and compared the results of this prediction to the experimental results of fragment screening. FTmap, run by masking the FAD binding site, identifies 15 clusters of small organic probes, ten of which can be grouped since they target the same cavity located at the dimeric interface of TR (Figure S3A-B). This result is in line with previous FTmap analyses (Revuelto et al, 2021; Teixeira et al, 2021) that identified this interfacial cavity as a druggable site for Trypanosoma and Leishmania spp. Moreover, a similar cavity is present in GR and is targeted by some noncompetitive or uncompetitive inhibitors such as xanthene (Savvides & Karplus, 1996) (Figure S3B). Three of these ten clusters (8, 5, 5 probes respectively) correspond to the binding site of fragment 71, located at the narrow entrance of the interfacial cavity (Figures S3B-C-D). No fragments have been found within the interfacial cavity, possibly because of poor accessibility due to the narrow entrance and the structural rigidity of the crystal packing. However, it is interesting to note that, besides hit 71, hit 90 in site 5 binds close to the cavity, adjacent to the location of xanthene in GR (Figure S3B). Other two clusters (6 and 2 probes respectively) are located in correspondence of the doorstop pocket (Figure S3E), although the site does not stand out as a significative druggable one. Intriguingly, no clusters are predicted to bind at the MBS, in line with the results of experimental fragment screening but in contrast with previously characterized inhibitors of TR.

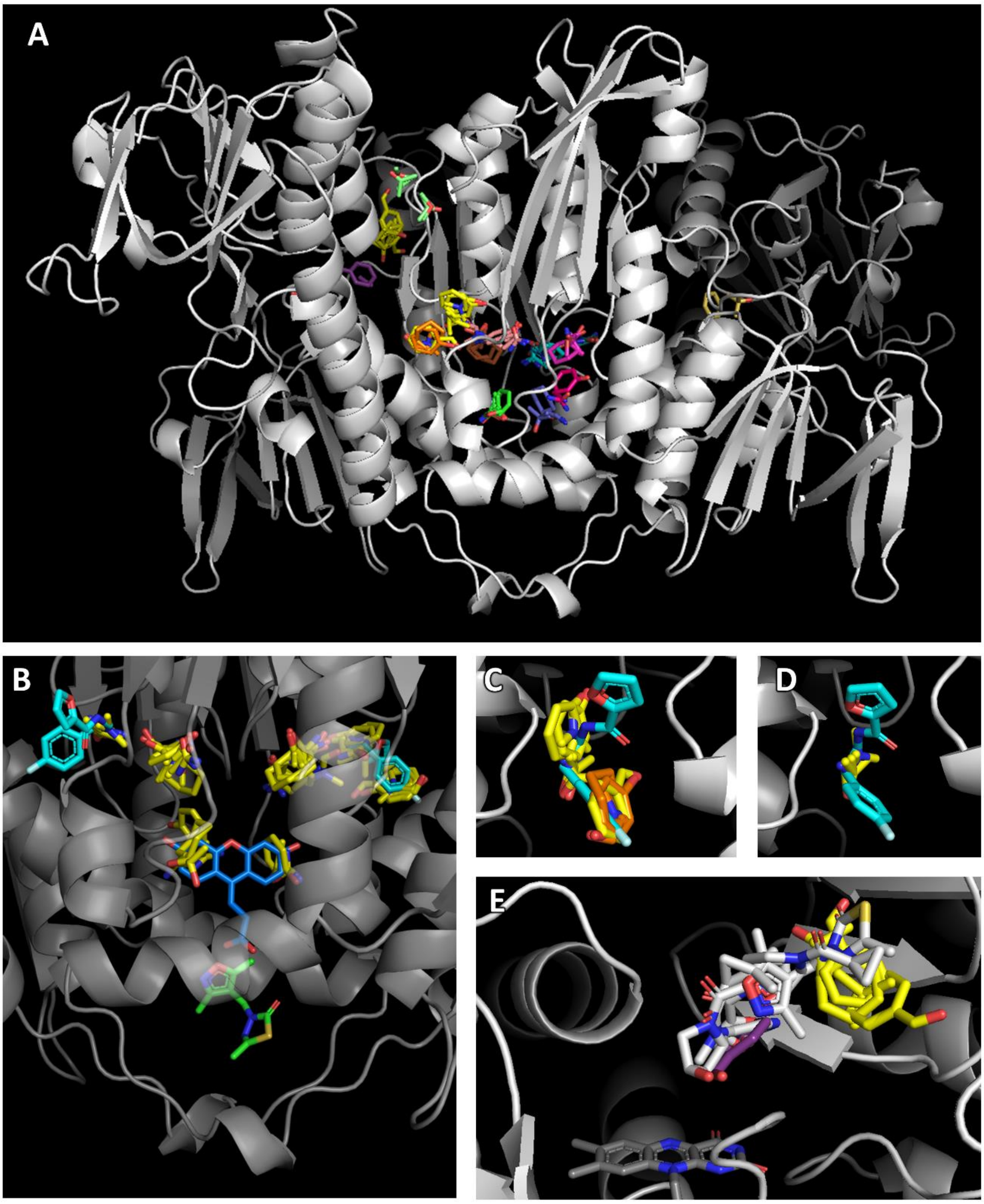

(A) Output of FTmap analysis, each probe cluster is colored differently. (B) Detail of the interfacial cavity. Clusters from FTmap analysis are colored yellow, fragment hits 71 and 90 are colored cyan and green, respectively. The xanthene ligand of GR (PDB ID: 1XAN) is colored blue. (C, D) Detail of clusters corresponding to hit 71 in subunits A and B of TbTR. (E) Predicted and experimental fragments located at the doorstop pocket. The experimental fragment hits are colored grey, the predicted ones are yellow and purple, FAD is shown as reference.

REFERENCE OF SUPPLEMENTARY MATERIAL

Kozakov, D., Grove, L. E., Hall, D. R., Bohnuud, T., Mottarella, S. E., Luo, L., Xia, B., Beglov, D. & Vajda, S. (2015) The FTMap family of web servers for determining and characterizing ligand-binding hot spots of proteins. *Nat Protoc*, 10(5), 733-55.

Revuelto, A., de Lucio, H., García-Soriano, J. C., Sánchez-Murcia, P. A., Gago, F., Jiménez-Ruiz, A., Camarasa, M. J. & Velázquez, S. (2021) Efficient Dimerization Disruption of. *J Med Chem*, 64(9), 6137-6160.

Savvides, S. N. & Karplus, P. A. (1996) Kinetics and crystallographic analysis of human glutathione reductase in complex with a xanthene inhibitor. *J Biol Chem*, 271(14), 8101-7.

Teixeira, O., Lacerda, P., Froes, T. Q., Nonato, M. C. & Castilho, M. S. (2021) Druggable hot spots in trypanothione reductase: novel insights and opportunities for drug discovery revealed by DRUGpy. *J Comput Aided Mol Des*, 35(8), 871-882.
